# Unilateral Absence of Musculocutaneous Nerve and Unusual Supply of Median Nerve – A Case Report

**DOI:** 10.15388/Amed.2024.31.2.22

**Published:** 2024-12-04

**Authors:** Dibakar Borthakur, Rajesh Kumar, Harshit Jain, Pooja Poddar, Monica Baxla

**Affiliations:** 1 Department of Anatomy, All India Institute of Medical Sciences, New Delhi, India; 2 Department of Anatomy, All India Institute of Medical Sciences, Patna, India

**Keywords:** Brachial plexus, coracobrachialis, median nerve, musculocutaneous nerve, Raktažodžiai: Brachialinis rezginys, korakobrachialinis raumuo, vidurinis nervas, muskulokutaninis nervas

## Abstract

**Introduction:**

Anatomical variations of the brachial plexus and its branches are frequently encountered. Absent musculocutaneous nerve with complete or partial takeover of function by median nerve is known. Such variation creates confusion in the interpretation of a clinical or electro diagnostic test for evaluation of peripheral nerve injury.

**Methods:**

Institutional guidelines for the use of human cadaver were followed. Routine dissection of the upper limbs for undergraduate medical teaching was performed in a 67-years-old male cadaver following standard methods. Relevant gross anatomical features of the variations were photographed.

**Results:**

The musculocutaneous nerve was absent on the left side. A direct small branch arising from the lateral cord supplied the coracobrachialis muscle. Median nerve supplied the biceps brachii and brachialis muscle and further continued as lateral cutaneous nerve of the forearm.

**Conclusion:**

The unilateral absence of musculocutaneous nerve with taking over of its function predominantly by the median nerve observed in the present case is indeed a unique situation to be aware of. Additionally the case exhibited independent supply to the coracobrachialis muscle directly from the lateral cord of the brachial plexus. It is proposed that this variation should be placed in a new subcategory in the existing classification system of the classification for musculocutaneous nerve variations.

## Abstract (it)

**Įvadas:**

Dažnai susiduriama su peties rezginio ir jo atšakų anatominėmis variacijomis. Žinomas raumeninio nervo nebuvimas ir visiškas arba dalinis vidurinio nervo funkcijos perėmimas. Tokie skirtumai sukelia painiavą interpretuojant klinikinį ar elektrodiagnostinį testą, skirtą periferinio nervo pažeidimui įvertinti.

**Metodai:**

Buvo laikomasi institucijos rekomendacijų dėl žmogaus lavono naudojimo. įprastinė viršutinių galūnių skrodimo operacija, skirta medicinos bakalauro studijoms, buvo atlikta 67 metų vyro lavono pagal standartinius metodus. Buvo nufotografuoti atitinkami bendrieji anatominiai variantų požymiai.

**Rezultatai:**

Kairėje pusėje raumeninio nervo nebuvo. Tiesioginė maža šaknelė, kylanti iš šoninio raumens, maitino korakobrachialinį raumenį. Vidurinis nervas maitino dvigalvį žasto raumenį ir brachialinį raumenį ir toliau tęsėsi kaip šoninis dilbio odos nervas.

**Išvados:**

Šiuo atveju nustatytas vienpusis raumeninio nervo nebuvimas, kai jo funkciją perima vidurinis nervas, iš tiesų yra unikali situacija, apie kurią reikia žinoti. Be to, šiuo atveju buvo pastebėtas nepriklausomas korakobrachialinio raumens aprūpinimas tiesiogiai iš šoninės peties rezginio dalies. Siūloma, kad šis variantas būtų įtrauktas į naują muskulokutaninio nervo variantų klasifikacijos sistemos subkategoriją.

## Introduction

The upper extremity is innervated by the brachial plexus, formed by the anterior primary rami of C5 to T1 spinal nerves with occasional contributions from either C4 or T2 spinal segments. Brachial plexus in classic description, comprises the roots (anterior rami of C5 to T1 spinal nerves), trunks, divisions, cords and branches of the plexus [1, 2]. The median nerve (MN) is formed anterior to the third part of axillary artery by union of the lateral root of MN (C5, C6, and C7) from the lateral cord and the medial root of the MN (C8, T1) from the medial cord and courses further towards the forearm without supplying any muscles of the arm [1,2]. The MN does not have any muscular branch in the arm except for an occasional branch to pronator teres muscle arising above the elbow joint. However vascular and articular branches are given off while it traverses the arm. In the forearm, it supplies the superficial flexor muscles except the flexor carpi ulnaris and medial half of the flexor digitorum profundus. In the hand, it provides motor innervations to the thenar muscles (superficial head of flexor pollicis brevis, abductor pollicis brevis and the opponens pollicis) and 1st two lumbricals. The MN supplies the skin of the lateral half of the palm, and lateral 3½ digits (complete palmar surface and dorsum of distal phalanges). Musculocutaneous nerve (MCN) arises from the lateral cord of the brachial plexus and has root value of C5, C6. In the arm, the MCN pierces the coracobrachialis (CRB), supplying it before its entry into the muscle and further runs between the biceps brachii and brachialis muscles while passing from medial to lateral aspect. It pierces the deep fascia just above the elbow and further continues as the lateral cutaneous nerve of the forearm (LCNF) to innervate the skin of the anterolateral aspect of the forearm [1,2]. Absent MCN with an incidence of 3.57–6.6% is an occasional finding in humans having several unanticipated clinical ramifications [3]. We are describing here a unique case of unilateral absence of MCN with concomitant variant branching pattern of MN. Awareness about the anatomical variation will help in the correct interpretation of unusual sensorimotor findings in clinical as well as in electro diagnostic tests.

## Case Report

**Fig. 1:**
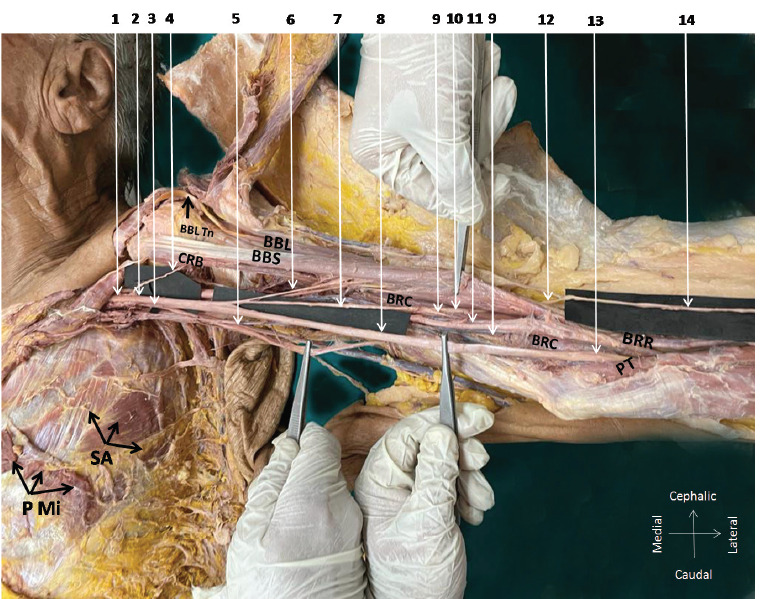
Dissected left brachial plexus and its branches in abducted left upper limb. 1 – lateral cord of brachial plexus, 2 – lateral root of the median nerve (MN), 3 – medial root of the MN, 4 – branch of the lateral cord directly supplying the coracobrachialis, 5 – common trunk of the medial cutaneous nerve of the arm and forearm, 6 – muscular branches to the biceps, 7 – mixed nerve branch sprouting from the median nerve, 8 – main trunk of the median nerve, 9 – median nerve branch supplying brachialis, 10 – median nerve descending after its muscle innervations, 11 – brachial artery with its venae comitantes, 12 – median nerve piercing the deep fascia, 13 –- MN at cubital fossa, 14 – lateral cutaneous nerve of the forearm, PMi – cut edges of pectoralis minor muscle, SA – serratus anterior muscle, CRB – coracobrachialis muscle, BBS – short head of biceps brachii muscle, BBL – long head of biceps brachii muscle, BBL Tn – tendon of biceps brachii long head, BRC – brachialis muscle, BRR – brachioradialis muscle, PT – pronator teres muscle

The present cadaveric case was observed in September, 2022 during routine undergraduate dissection performed in a 67-year-old male cadaver. The authors hereby confirm that every effort was made to comply with the local and international ethical guidelines and laws concerning the use of human cadaver. The upper limbs of both sides were dissected carefully employing the standard dissection methods. On the left side, the MCN was absent and CRB muscle was supplied by an ultrathin nerve arising from the lateral cord just before the origin of lateral root of the MN. Additional innervations to CRB were absent. The lateral root and the medial root of the MN united to form the main trunk of the MN anterior to the 3rd part of the axillary artery. Soon after formation, the MN bifurcated into two branches. The thin lateral branch supplied the biceps brachii, brachialis and then further continued as the LCNF (Fig. 1). The thick medial branch mimicked a typical MN and descended to the forearm. No other associated anomalies of neurovascular structures of the left upper limb were noted. The right upper limb did not have any anatomical variations. No evidence of atrophy of flexor compartment muscles of the left arm supplied by MN was observed when compared with flexor compartment muscles of the right arm which was supplied by MCN.

## Discussion

The upper limb bud appears by the end of the fourth week. During the fifth week of gestation, under the influence of *homeobox D* genes, the limb muscles develop. Peripheral nerves from the developing brachial plexus grow into the mesenchyme of the upper limb by forming growth cones of motor axons which under the influence of factors such as *brain derived nerve growth factor (BDNF), ephrins, neutrin-1, neutrin-2, semaphorins*, etc. grow, differentiate and form accurate neural connections to innervate the predestined target [4]. Neuronal cell bodies at the dorsal root ganglia (DRG) project peripheral axons to the developing limb bud under the control of *runt-related transcription factor-3 (RUNX3)*. Peripheral innervations is also regulated by transcription factor *neurotrophin-3 (NT3)* secreted by the limb mesenchyme with support from the simultaneously developing motor neuron axons [5]. It is believed that deviation in the normal molecular signalling pathway results in anatomic variation of brachial plexus.

Several variations of the MCN including absent MCN are known [4, 6–10]. The typical MCN supplying a CRB was not observed on the left side in this case. Instead a direct tiny ultrathin branch arising from the lateral cord of the brachial plexus supplied the left CRB [8, 11]. Unilateral absence of MCN with variant MN has been noted in studies across different population groups [4, 9, 12–15]. Few studies reported bilateral absence of MCN with variations in the MN [6–8, 11, 16]. Most of the information about the absent MCN with taking over of functions by MN is obtained from case reports. Among the few cadaveric studies, a prevalence of absent MCN has been documented to be 3.57% in the Spanish population [6]. In contrast, a study in the Indian population found 6.6% prevalence of absent MCN [16]. However, the results of the studies conducted on relatively small sample sizes may not represent the actual prevalence. Several researchers classified MCN into distinct subtypes depending on criteria such as MCN piercing the CRB and the extent of communication with the MN [6, 17]. The present case when compared with the existing classification system closely resembles the type *f.1 sub-category of Guttenberg’s* and *type V of Le Minor’s* classification system [6, 10]. We propose that a new taxonomic subcategory should be introduced to precisely denote this variation of MCN in the existing classification system within the category of absent MCN. The anatomic variations of the MCN and MN presented here are important for evaluating a case of suspected MN injury. Clinically, weakness of the flexor muscles of the arm along with sensory deficit in the anterolateral forearm might lead to erroneous conclusion of concomitant MCN injury. Furthermore, cases of lateral cord or MCN injury can be easily missed on clinical examination because of takeover of function by the variant MN.

## Conclusion

We propose that a new taxonomic subcategory should be introduced to precisely denote this variation of musculocutaneous and median nerve in the existing classification system within the category of absent musculocutaneous nerve. Awareness of such variation is clinically relevant during evaluation of brachial plexus injury.

## Limitation

Although the present case reports a unique variation in the brachial plexus, prevalence and clinical relevance of the same should be evaluated in the context of the findings obtained from a well designed study with adequate sample size.
